# ^18^F-FDG PET or PET/CT in detecting high-grade transformation of chronic lymphocytic leukaemia and indolent lymphomas: a systematic review and meta-analysis

**DOI:** 10.1093/bjr/tqaf028

**Published:** 2025-02-11

**Authors:** Osher N Y Lee, John Kuruvilla, David C Hodgson, Patrick Veit-Haibach, Ur Metser

**Affiliations:** Edinburgh Medical School, College of Medicine and Veterinary Medicine, University of Edinburgh, Edinburgh EH16 4SB, United Kingdom; Department of Medical Oncology and Haematology, Princess Margaret Cancer Centre, University of Toronto, Toronto, ON M5G 2M9, Canada; Radiation Medicine Program, Princess Margaret Cancer Centre, University Health Network, Toronto, ON M5G 2M9, Canada; University Medical Imaging Toronto; Department of Medical Imaging, University Health Network, Sinai Health Systems & Women’s College Hospital, University of Toronto, Toronto, ON M5G 2M9, Canada; University Medical Imaging Toronto; Department of Medical Imaging, University Health Network, Sinai Health Systems & Women’s College Hospital, University of Toronto, Toronto, ON M5G 2M9, Canada

**Keywords:** fluorodeoxyglucose F18, positron emission tomography computed tomography, lymphoma, high-grade transformation, Richter’s transformation

## Abstract

**Objectives:**

To evaluate the diagnostic accuracy of ^18^F-FDG positron emission tomography (PET) or PET/computed tomography (CT) in detecting histological transformation (HT) of indolent lymphomas.

**Methods:**

A systematic search of articles up to July 2024 was performed in Embase and Medline. Eligible studies included adults with histologically proven indolent lymphoma, ^18^F-FDG PET or PET/CT as the index test, and sufficient data to assess diagnostic performance. Summary receiver operating characteristic curves were plotted using a bivariate model to estimate diagnostic accuracy with area under the curve (AUC).

**Results:**

Fifteen studies with 1307 participants were included. Ten studies assessed PET ability to detect Richter’s transformation, and 5 studies focused on HT in follicular lymphoma and other subtypes. A meta-analysis of the former showed pooled sensitivity of 0.90 (95% CI, 0.84-0.93) and specificity of 0.54 (95% CI, 0.28-0.77) when using a maximum standardized uptake value (SUV_max_) threshold of around 5. AUC was 0.89. Pooled sensitivity was 0.74 (95% CI, 0.54-0.87), and specificity was 0.84 (95% CI, 0.67-0.93) when using an SUV_max_ threshold of around 10. Area under the curve was 0.84. For detecting HT in follicular lymphoma, thresholds were found higher than those for Richter’s transformation.

**Conclusions:**

^18^F-FDG PET or PET/CT demonstrates good diagnostic accuracy to detect Richter’s transformation, best when employing SUV_max_ ≥ 5. SUV_max_ thresholds may be limited in discriminating follicular lymphoma from HT, and alternatives should be sought.

**Advances in knowledge:**

If biopsy is feasible, SUV_max_ ≥ 5 can guide biopsy in patients with clinically suspicious Richter’s transformation. If biopsy is infeasible, SUV_max_ ≥ 10 can better identify HT and guide patient management.

## Introduction

Non-Hodgkin lymphomas (NHLs) are a group of haematological neoplasms that constitute nearly 3% of cancer diagnoses and deaths.^1^ Diffuse large B cell lymphoma (DLBCL) is the most prevalent NHL, while follicular lymphoma (FL) accounts for most cases of indolent lymphomas, followed by marginal zone lymphoma (MZL) and chronic lymphocytic leukaemia (CLL)/small lymphocytic lymphoma (SLL).^1^ Indolent NHLs can undergo histological transformation (HT) to aggressive histopathologies, which occur in approximately 3% of cases annually.^2^^,^^3^ For CLL/SLL, Richter’s transformation is the term adopted to describe the transformation to a more aggressive form of lymphoma, typically DLBCL. Histological transformation can present clinically with non-specific features, including rapidly growing nodes, extranodal site involvement, systemic B symptoms, hypercalcemia, and elevated serum lactate dehydrogenase.^2^ These features prompt further imaging and biopsy to confirm HT.

Although histopathological analysis on biopsy remains the gold standard for the diagnosis of NHLs, positron emission tomography (PET) with the radiotracer ^18^F-fluorodeoxyglucose (FDG) has played an increasing clinical role in the staging and monitoring of NHLs.^4^ In comparison to anatomical techniques such as computed tomography (CT) or magnetic resonance imaging (MRI), ^18^F-FDG PET benefits from the ability to recognize metabolic changes before structural changes become apparent. High FDG uptake as expressed by the standardized uptake value (SUV) can be used to identify optimal sites from which biopsies can be taken.^5^

Various studies report that FDG uptake on PET is lower in indolent lymphomas and higher in aggressive lymphomas.^6^^,^^7^ Transformed lymphomas have also been shown to present with uptake similar to values seen in DLBCL.^8^ However, evidence surrounding the diagnostic ability of ^18^F-FDG PET/CT to distinguish cases with and without high-grade transformation is incomplete. Although different maximum SUV (SUV_max_) cut-offs have been proposed in the literature,^9^^,^^10^ FDG uptake in indolent lymphomas can vary and be heterogeneous.^11^^,^^12^ Follicular lymphoma in particular can often demonstrate intense FDG uptake, which could overlap with DLBCL or HT and reduce the specificity of ^18^F-FDG PET in diagnosing HT. This may pose a challenge in identifying reasonable thresholds to discriminate indolent lymphomas from HT_._ The current systematic review and meta-analysis therefore aims to evaluate the diagnostic accuracy of ^18^F-FDG PET or PET/CT for the detection of HT in different histological indolent lymphoma subtypes, especially CLL/SLL and FL, and to determine SUV_max_ cut-offs that might be used to guide subsequent management.

## Methods

This review follows the Preferred Reporting Items for Systematic Reviews and Meta-Analyses of Diagnostic Test Accuracy Studies (PRISMA-DTA) statement.^13^ A review protocol was registered in the PROSPERO database (CRD42024514567).

### Search strategy

A literature search of articles published up to July 2024 was conducted using the Embase and Medline databases. The terms “positron emission tomography”, “indolent lymphoma”, “non-Hodgkin lymphoma”, “follicular lymphoma”, “chronic lymphocytic leukaemia”, “small lymphocytic lymphoma”, “transform*”, and “Richter*” and their respective abbreviations were used. No additional filters were applied. Citation searching was performed in included reports to identify suitable studies not found in the database search.

### Study selection

The inclusion criteria are as follows: the study must be an observational study, include adults with histologically proven indolent lymphoma suspicious of transformation, use ^18^F-FDG PET or PET/CT as the index test, and have sufficient data to generate a 2 × 2 table to assess sensitivity and specificity of PET for detecting HT in lymphoma patients at a given SUV_max_ threshold. Patients presenting with aggressive lymphoma without a previous diagnosis of indolent lymphoma were excluded. Case reports and studies with < 10 patients were excluded. Our resources allowed for searching of meeting abstracts. These were considered eligible for inclusion, which is advocated by Scherer and Saldanha^14^ for topics with inconclusive and limited evidence. In studies with overlapping populations, the most recent eligible publication was chosen.

### Data extraction

Information on study design, cohort characteristics (sample size, age, male to female ratio, patients by lymphoma histopathological subtype, number of patients with HT), and technical scanner aspects (type of scan, FDG dose, time between FDG administration and scan, PET interpretation criteria) were extracted independently from eligible studies. Median or mean and range of SUV_max_ uptake for those without HT and with HT were retrieved. Characteristics were labelled as not available (NA) if not reported.

For each reported SUV_max_ cut-off, true-positive (TP), false-positive (FP), false-negative (FN), and true-negative (TN) values on a per-patient assessment for ^18^F-FDG PET/CT to detect HT were retrieved from studies to determine sensitivity, specificity, positive predictive value (PPV), and negative predictive value (NPV). Biopsy was considered the gold standard to evaluate the diagnostic ability of PET/CT. Findings were separated by lymphoma histology to outline PET/CT performance in each subtype. In studies including a population of mixed histopathologies with available patient-level data for histology and SUV_max_ uptake, we calculated the most optimal SUV_max_ threshold for individual subtypes by performing receiver operating characteristic (ROC) curve analyses.

### Quality assessment

Quality assessment was performed using the Quality Assessment of Diagnostic Accuracy Studies Two (QUADAS-2) Tool.^15^ Risk of bias and applicability concerns were assessed in each of the 4 domains: patient selection, index test, reference standard, flow, and timing.

### Statistical analysis

Meta-analysis was conducted to determine the diagnostic accuracy of ^18^F-FDG PET or PET/CT to detect HT in different subtypes of indolent lymphoma. Bivariate analysis using the random effects model was performed to estimate pooled statistics for sensitivity and specificity, which were visualized using forest plots. Summary ROC (SROC) curves were then plotted using the model described by Chu and Cole^16^ to calculate the associated area under the curve (AUC) and interpret overall diagnostic performance. SROC curves were drawn with the x-axis as the FP rate (1−specificity) and the y-axis as sensitivity. Heterogeneity among studies was assessed using the *I^2^* statistic. Heterogeneity for the SROC curve was assessed using the correlation coefficient of sensitivity and specificity. A value was considered high if greater than 0.^17^

Statistical investigations were conducted separately for each lymphoma subtype to characterize SUV uptake, which can vary among different histopathologies.^11^^,^^12^ The data were synthesized and grouped by similar SUV_max_ thresholds to identify a cut-off range that is most effective for detecting HT. As recommended,^14^ a separate analysis excluding data from meeting abstracts would be conducted if findings were unique. All statistical analyses were performed using Stata version 18.5.

## Results

### Literature search and study selection

**Figure 1.**
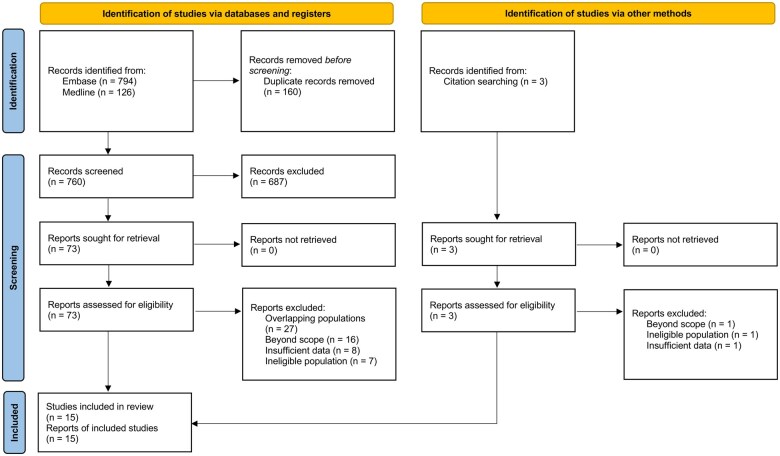
PRISMA flow for selection of studies. A total of 760 unique studies were identified via databases and registers, and an additional 3 studies were identified from citation searching. Fifteen studies were eligible for systematic review and meta-analysis.

Of the 920 records identified, 15 studies were included in the systematic review. Ten studies were eligible for meta-analysis. Abstract screening excluded 687 records, of which most were case reports, reviews, and studies beyond the scope of this study. Full-text screening excluded 58 reports, of which most were conference abstracts overlapping with an associated journal publication. Details of the study selection are depicted by the PRISMA flow^18^ in Figure 1.

### Study characteristics and SUV_max_ values

Details on study design, cohort characteristics, and technical scanner aspects of each study are summarized in Table 1. Eleven studies adopted a retrospective design, and 3 adopted a prospective design. Ten studies focused on CLL/SLL, 2 focused on FL, and 3 included a mixed population of patients with various lymphoma subtypes. A total of 1307 patients were included, of which 1012 (77%) had CLL/SLL, 249 (19%) had FL, and 46 (4%) had other histology. Of the 1307 patients, 360 (28%) experienced HT. Twelve studies used PET/CT to detect lymphoma transformation, 2 used PET only, and 1 study used a combination of both PET/CT and PET. Only 5 studies provided information on FDG dose and its uptake time. All studies used a combination of visual and semiquantitative PET parameters to interpret PET findings. There were 3 meeting abstracts included in our analysis.

**Table 1.** Characteristics of the included studies.

First author	Year	Study design	Patients included in final analysis	Patients by initial iNHL subtype	M:F ratio	Median age (range)	Patients with HT	Device	Mean FDG dose (MBq)	Time between FDG administration and scan (min.)	PET interpretation criteria
Albano et al^21^	2021	Retrospective cohort	80	CLL/SLL = 80	58:22	61.5 (27-83)	18 (22.5%)	PET/CT	3.5-4.5 kg^−1^	60	Visual, semiquantitative (SUV_max_, SUV_lbm_, SUV_bsa_, SUV_L/L_, SUV_L/BP_, MTV, TLG)
Bodet-Milin et al^19^	2008	Prospective cohort	38	FL = 23 CLL/SLL = 10 MZL = 3 WM = 2	16:22	63 (38-81)	17 (44.7%)	PET/CT	NA	NA	Visual, semiquantitative (SUV_max_, SUV_max_ gradient)
Bruzzi et al^9^	2006	Retrospective cohort	37	CLL/SLL = 37	26:11	61^a^ (40-82)	11 (29.7%)	PET/CT	555	60	Visual, semiquantitative (SUV_max_)
Du^23^^,^^a^	2015	Retrospective cohort	56	CLL/SLL = 56	NA	NA	31 (55.4%)	PET/CT	NA	NA	NA
Falchi et al^40^	2014	Retrospective cohort	332	CLL/SLL = 332	218:114	68 (31-85)	95 (28.6%)	PET/CT and PET	NA	NA	Visual, semiquantitative (SUV_max_)
Mato et al^41^	2019	Prospective cohort	35	CLL/SLL = 35	NA	NA	7 (20.0%)	PET/CT	NA	NA	Visual, semiquantitative (SUV_max_)
Mauro et al^42^	2015	Retrospective cohort	90	CLL/SLL = 90	65:25	61.2 (31-81)	17 (18.9%)	PET/CT	NA	NA	Visual, semiquantitative (SUV_max_)
Michallet et al^10^	2016	Retrospective cohort	240	CLL/SLL = 240	94:146	62^a^ (21-91)	24 (10.0%)	PET/CT	NA	NA	Visual, semiquantitative (SUV_max_)
Obeid et al^28^	2023	Retrospective case-control	103^c^	FL = 83 Non-FL = 23	56:50	HT: 57 (34-73) Control: 50 (29-80)	50 (48.5%)	PET/CT	444-666	45-60	Visual, semiquantitative (SUV_max_, MTV, TLG)
Rajamäki et al^25^	2023	Retrospective cohort	63	FL = 63	29:34	HT: 68 (59-88) No HT: 60 (29-87)	7 (11.1%)	PET/CT	3.43 kg^−1^	60	Visual, semiquantitative (SUV_max_)
Wai et al^26^	2024	Retrospective cohort	84^c^	FL = 54 CLL/SLL = 14 MZL = 14 Other = 4	NA	NA	31 (36.9%)	PET	NA	NA	Visual, semiquantitative (SUV_max_, SUV_mean_, MTV, TLG)
Wang et al^43^	2020	Retrospective cohort	54	CLL/SLL = 54	43:11	67 (43-81)	25 (46.3%)	PET	NA	NA	Visual, semiquantitative (SUV_max_)
Wondergem et al^24^	2015	Prospective case-control	26	FL = 26	NA	57 (34-80)	9 (34.6%)	PET/CT	185	60	Visual, semiquantitative (SUV_max_)
Zhang et al^22^^,^^b^	2016	NA	12	CLL/SLL = 12	8:4	NA	4 (33.3%)	PET/CT	NA	NA	NA
Zheng et al^20^^,^^b^	2020	Retrospective cohort	52	CLL/SLL = 52	36:16	56 (16-81)	14 (26.9%)	PET/CT	NA	NA	NA

a Mean.

b Meeting abstracts.

c Excluding cases without available PET data.

Abbreviations: aCLL/SLL: histologically aggressive CLL/SLL; CLL: chronic lymphocytic leukaemia; FL: follicular lymphoma; HT: histological transformation; iCLL/SLL: histologically indolent CLL/SLL; iNHL: indolent non-Hodgkin lymphoma; MTV: metabolic tumour volume; MZL: marginal zone lymphoma; NA: not available; SLL: small lymphocytic lymphoma; SUV_bsa_: SUV_max_ corrected for body surface area; SUV_max_: maximum standardized uptake value; SUV_mean_: average SUV; SUV_lbm_: SUV_max_ corrected for lean body mass; SUV_L/L_: SUV_max_ lesion to liver ratio; SUV_L/BP_: SUV_max_ lesion to blood-pool ratio; TLG: total lesion glycolysis; WM: Waldenström macroglobulinemia.

Table 2 shows the median or mean SUV_max_ uptake in patients with HT versus patients without HT. All studies reported a greater average uptake in individuals who experienced HT than individuals who did not. Studies focusing on FL or mixed histopathologies tended to present with greater median or mean SUV_max_ than those focusing on CLL/SLL.

**Table 2.** Comparison of SUVmax between cases with and without histological transformation.

First author	Patients by initial iNHL subtype	Median SUV_max_ of cases without HT (range)	Median SUV_max_ of cases with HT (range)
Albano D	CLL/SLL = 80	5^a^ (1.3-22.4)	15.7^a^ (1.9-56)
Bodet-Milin C	FL = 23 CLL/SLL = 10 MZL = 3 WM = 2	8.6 (1.7-17.0)	18.5 (11.7-41.2)
Bruzzi JF	CLL/SLL = 37	4.5^a^ (2-7.9)	17.6^a^ (7.4-39.4)
Du Y	CLL/SLL = 56	NA	NA
Falchi L	CLL/SLL = 332	iCLL/SLL: 3.7 (0-14.3) aCLL/SLL: 6.8 (0-37.8)	17.6 (0-56.3)
Mato AR	CLL/SLL = 35	NA	12 (0.5-22)
Mauro FR	CLL/SLL = 90	3.5 (1-17.5)	10 (3.5-36)
Michallet AS	CLL/SLL = 240	iCLL/SLL: 2.2 (0-3.4) aCLL/SLL: 4.5 (1-11)	12.9 (5-27)
Obeid JP	FL = 83 Non-FL = 23	8.7^a^ (1.7-22.6)	22^a^ (4.5-57.3)
Rajamäki A	FL = 63	NA	27.1 (10.5-34.9)
Wai SH	FL = 54 CLL/SLL = 14 MZL = 14 Other = 4	10.7 (7.1-15.3^b^)	13.8 (10.6-26.3^b^)
Wang Y	CLL/SLL = 54	6.4 (1.8-12.5)	11.3 (4.6-24)
Wondergem MJ	FL = 26	10.9 (5.3-21)	22 (14.7-42.2)
Zhang J	CLL/SLL = 12	NA	NA
Zheng X	CLL/SLL = 52	NA	NA

a Mean.

b Interquartile range.

Abbreviations: aCLL/SLL: histologically aggressive CLL/SLL; CLL: chronic lymphocytic leukaemia; FL: follicular lymphoma; HT: histological transformation; iCLL/SLL: histologically indolent CLL/SLL; iNHL: indolent non-Hodgkin lymphoma; MZL: marginal zone lymphoma; NA: not available; SLL: small lymphocytic lymphoma; SUV_max_: maximum standardized uptake value; WM: Waldenström macroglobulinemia.

### Quality assessment

**Figure 2.**
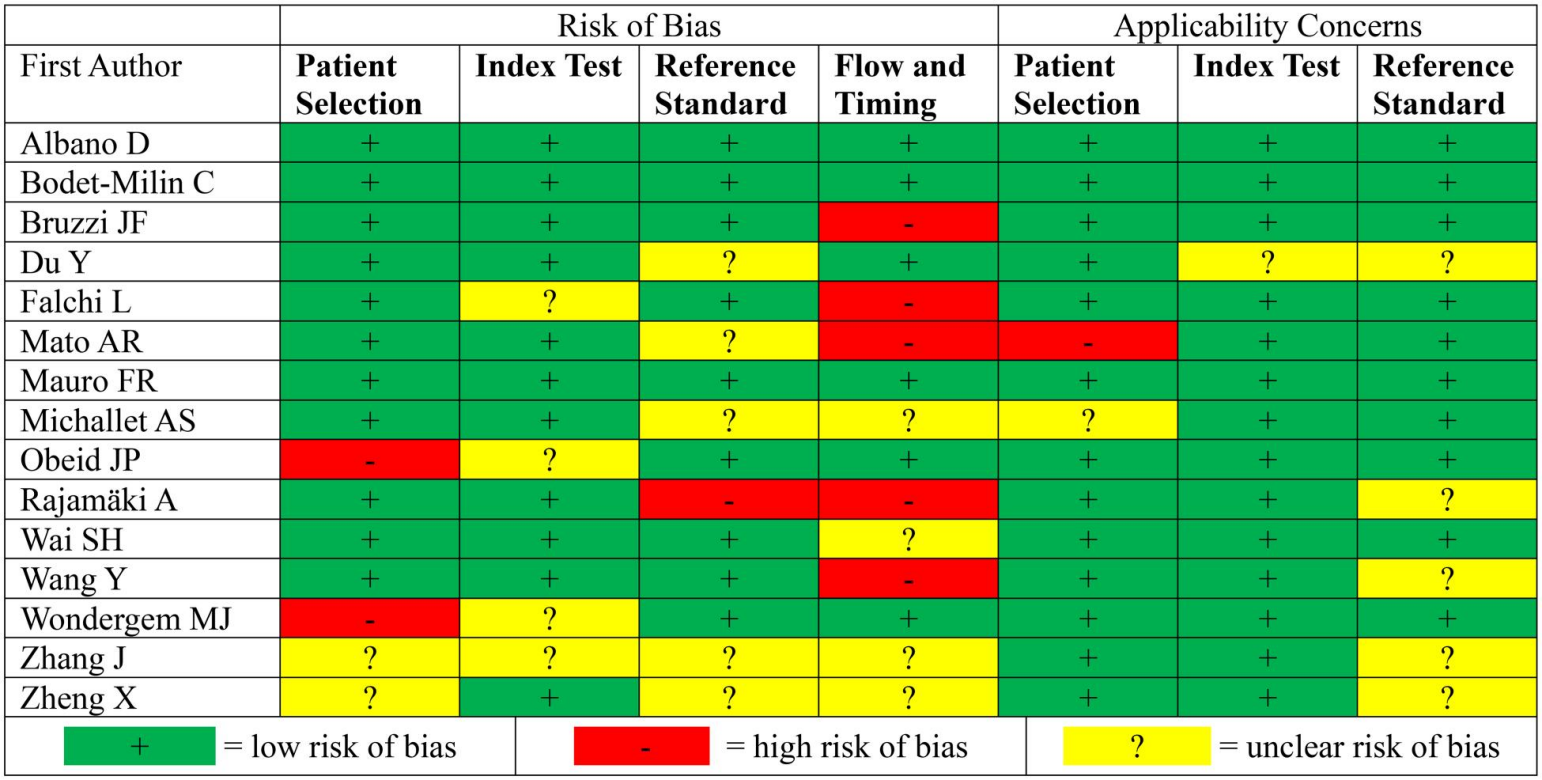
QUADAS-2 results for risk of bias and applicability concerns.

Figure 2 shows the results of the risk of bias and applicability concerns of the included studies using the QUADAS-2 tool. Studies that were high risk for patient selection adopted a case-control design where patients were split into control and HT groups before PET scanning. Studies that were high risk for flow and timing, or whether all patients underwent the same diagnostics within an appropriate period, most often resulted from long intervals between the index test (^18^F-FDG PET or PET/CT) and reference standard (biopsy). There was unclear risk of bias for the index test and reference standard for studies that did not clearly report which test was performed first or the implementation of blinding. Studies had minimal concerns regarding applicability.

### Diagnostic accuracy of ^18^F-FDG PET/CT

A summary of the diagnostic performance of ^18^F-FDG PET/CT to detect HT in CLL/SLL, FL, and mixed populations is displayed in Tables 3-5, respectively. Of the mixed population studies, which in total included 225 patients, only Bodet-Milin et al^19^ had available patient-level data specific to different lymphoma subtypes. ROC analysis showed that an SUV_max_ ≥ 11 for CLL/SLL and SUV_max_ ≥ 14 for FL were most optimal for detecting HT in their cohort.

**Table 3.** Diagnostic accuracy of ^18^F-FDG PET or PET/CT to detect Richter’s transformation in chronic lymphocytic leukaemia/small lymphocytic lymphoma.

First author	SUV_max_ cut-off	TP	FP	FN	TN	Sensitivity (95% CI)	Specificity (95% CI)	PPV (95% CI)	NPV (95% CI)
Albano D	9	12	6	6	56	0.67 (0.41-0.87)	0.90 (0.80-0.96)	0.67 (0.41-0.87)	0.90 (0.80-0.96)
Bodet-Milin C	5	4	3	0	3	1.00 (0.40-1.00)	0.50 (0.12-0.88)	0.57 (0.18-0.90)	1.00 (0.29-1.00)
	10	4	1	0	5	1.00 (0.40-1.00)	0.83 (0.36-1.00)	0.80 (0.28-0.99)	1.00 (0.48-1.00)
	11	4	0	0	6	1.00 (0.40-1.00)	1.00 (0.54-1.00)	1.00 (0.40-1.00)	1.00 (0.54-1.00)
Bruzzi JF	5	10	9	1	17	0.91 (0.59-1.00)	0.65 (0.44-0.83)	0.53 (0.29-0.76)	0.94 (0.73-1.00)
Du Y	NA	30	2	1	23	0.97 (0.83-1.00)	0.92 (0.74-0.99)	0.94 (0.79-0.99)	0.96 (0.79-1.00)
Falchi L	5	84	125	11	112	0.88 (0.80-0.94)	0.47 (0.41-0.54)	0.40 (0.33-0.47)	0.91 (0.85-0.95)
Mato AR	5	5	27	2	1	0.71 (0.29-0.96)	0.04 (0.00-0.18)	0.16 (0.05-0.33)	0.33 (0.01-0.91)
	10	5	14	2	14	0.71 (0.29-0.96)	0.50 (0.31-0.69)	0.26 (0.09-0.51)	0.88 (0.62-0.98)
	11	5	11	2	17	0.71 (0.29-0.96)	0.61 (0.41-0.78)	0.31 (0.11-0.59)	0.89 (0.67-0.99)
	12	4	9	3	19	0.57 (0.18-0.90)	0.68 (0.48-0.84)	0.31 (0.09-0.61)	0.86 (0.65-0.97)
Mauro FR	5	15	24	2	49	0.88 (0.64-0.99)	0.67 (0.55-0.78)	0.38 (0.23-0.55)	0.96 (0.87-1.00)
Michallet AS	10	22	11	2	205	0.92 (0.73-0.99)	0.95 (0.91-0.97)	0.67 (0.48-0.82)	0.99 (0.97-1.00)
Wang Y	5	24	23	1	6	0.96 (0.80-1.00)	0.21 (0.08-0.40)	0.51 (0.36-0.66)	0.86 (0.42-1.00)
	9	18	8	7	21	0.72 (0.51-0.88)	0.72 (0.53-0.87)	0.69 (0.48-0.86)	0.75 (0.55-0.89)
	10	14	7	11	22	0.56 (0.35-0.76)	0.76 (0.56-0.90)	0.67 (0.43-0.85)	0.67 (0.48-0.82)
Zhang J	5	3	0	1	8	0.75 (0.19-0.99)	1.00 (0.63-1.00)	1.00 (0.29-1.00)	0.89 (0.52-1.00)
Zheng X	6.4	13	6	1	32	0.93 (0.66-1.00)	0.84 (0.69-0.94)	0.68 (0.43-0.87)	0.97 (0.84-1.00)

Abbreviations: FN: false negative; FP: false positive; NA: not available; NPV: negative predictive value; PPV: positive predictive value; SUV_max_: maximum standardized uptake value; TN: true negative; TP: true positive.

**Table 4.** Diagnostic accuracy of ^18^F-FDG PET/CT to detect high-grade histological transformation in follicular lymphoma.

First author	SUV_max_ cut-off	TP	FP	FN	TN	Sensitivity (95% CI)	Specificity (95% CI)	PPV (95% CI)	NPV (95% CI)
Bodet-Milin C	14	9	2	1	11	0.90 (0.55-1.00)	0.85 (0.55-0.98)	0.82 (0.48-0.98)	0.92 (0.62-1.00)
Rajamäki A	26.5	6	0	1	56	0.86 (0.42-1.00)	1.00 (0.94-1.00)	1.00 (0.54-1.00)	0.98 (0.91-1.00)
Wondergem MJ	14.5	9	3	0	14	1.00 (0.66-1.00)	0.82 (0.57-0.96)	0.75 (0.43-0.95)	1.00 (0.77-1.00)

Abbreviations: FN: false negative; FP: false positive; NPV: negative predictive value; PPV: positive predictive value; SUV_max_: maximum standardized uptake value; TN: true negative; TP: true positive.

**Table 5.** Diagnostic accuracy of ^18^F-FDG PET or PET/CT to detect high-grade histological transformation in all types of indolent lymphoma.

First author	SUV_max_ cut-off	TP	FP	FN	TN	Sensitivity (95% CI)	Specificity (95% CI)	PPV (95% CI)	NPV (95% CI)
Bodet-Milin C	14	16	1	1	20	0.94 (0.71-1.00)	0.95 (0.76-1.00)	0.94 (0.71-1.00)	0.95 (0.76-1.00)
Obeid JP	10	44	14	6	39	0.88 (0.76-0.95)	0.74 (0.60-0.85)	0.76 (0.63-0.86)	0.87 (0.73-0.95)
Wai SH	12	22	22	9	31	0.71 (0.52-0.86)	0.58 (0.44-0.72)	0.50 (0.35-0.65)	0.78 (0.62-0.89)
	15	14	14	17	39	0.45 (0.27-0.64)	0.74 (0.60-0.85)	0.50 (0.31-0.69)	0.70 (0.56-0.81)
	25	8	2	23	51	0.26 (0.12-0.45)	0.96 (0.87-1.00)	0.80 (0.44-0.97)	0.69 (0.57-0.79)

Abbreviations: FN: false negative; FP: false positive; NPV: negative predictive value; PPV: positive predictive value; SUV_max_: maximum standardized uptake value; TN: true negative; TP: true positive.

#### Chronic lymphocytic leukaemia/small lymphocytic lymphoma

Meta-analysis was performed to identify PET or PET/CT ability to detect Richter’s transformation. There were 2 clear groups of SUV_max_ thresholds used among the studies: either “moderate” (5-6.4) or “high” (9-12). In the moderate group, findings using SUV_max_ ≥ 5 was most prominent and included into the analysis. Zheng et al^20^ only reported a cut-off of 6.4, so this threshold was chosen for their data. In the high group, findings using SUV_max_ ≥ 10 were most prominent and included into the analysis. Albano et al^21^ only reported a cut-off of 9, so this threshold was chosen for their data.

**Figure 3.**
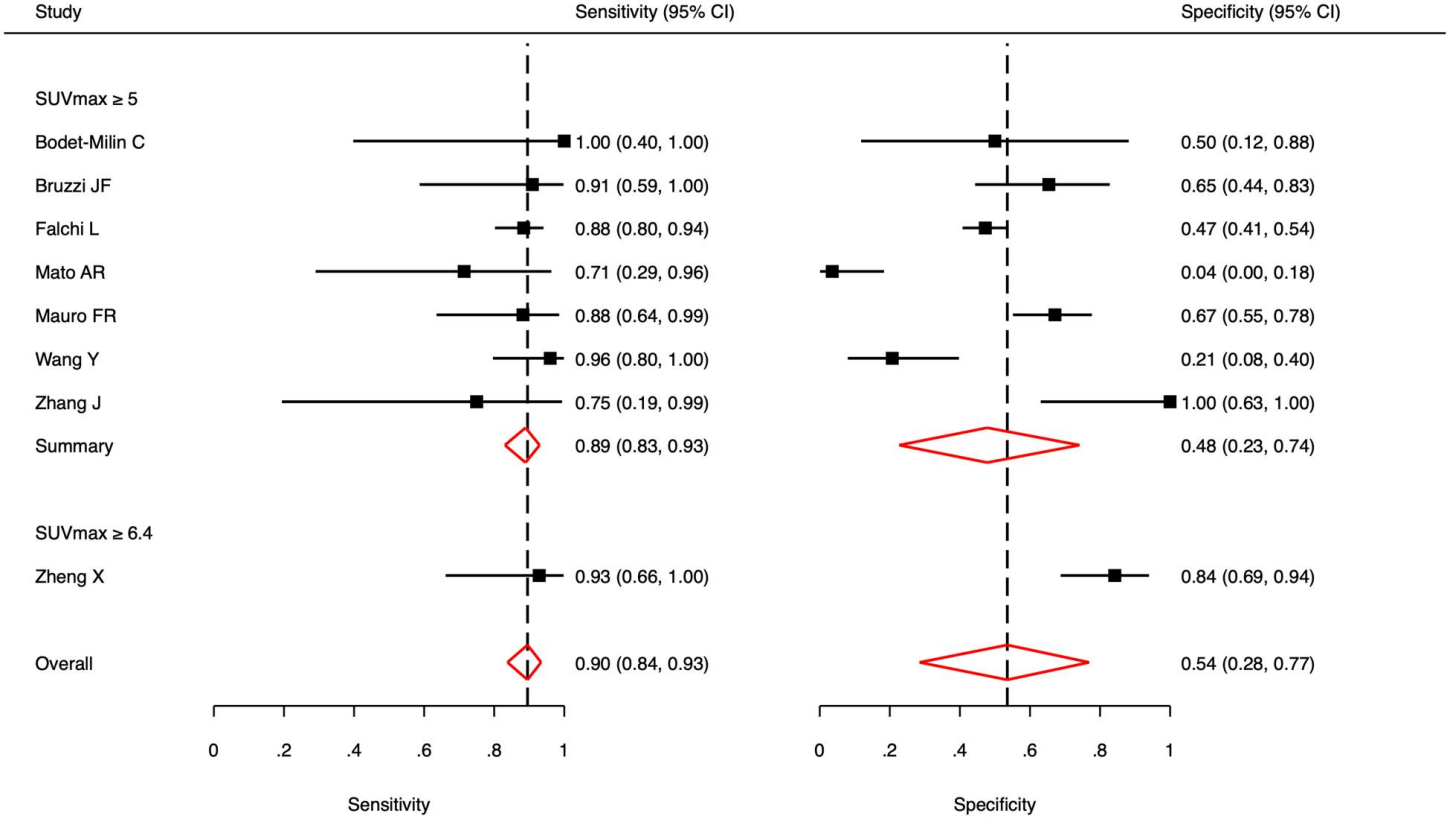
Forest plot of pooled sensitivity and specificity of ^18^F-FDG PET or PET/CT to detect Richter’s transformation using a moderate SUV_max_ threshold. Heterogeneity (*I^2^*) was 0% for sensitivity and 83% for specificity.

**Figure 4.**
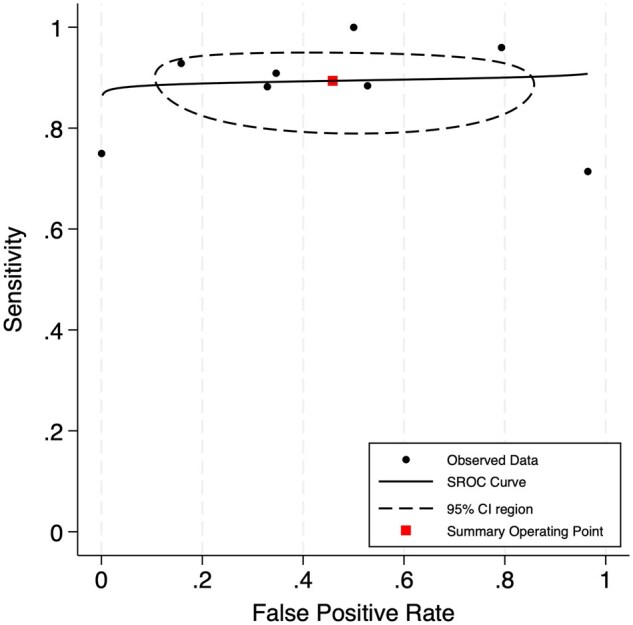
Summary receiver operating characteristic (SROC) curve illustrating the diagnostic accuracy of ^18^F-FDG PET or PET/CT to detect Richter’s transformation using a moderate SUV_max_ threshold. Area under the curve (AUC) was 0.89.

Forest plots for sensitivity and specificity of the moderate SUV_max_ group are depicted in Figure 3. The pooled sensitivity was 0.90 (95% CI, 0.84-0.93) with heterogeneity (*I^2^*) low at 0%. The pooled specificity was 0.54 (95% CI, 0.28-0.77) with heterogeneity (*I^2^*) high at 83%. The bivariate analysis is illustrated by the SROC curve in Figure 4. AUC was 0.89, indicating good diagnostic accuracy. The correlation coefficient between sensitivity and specificity was −0.044, which suggests low heterogeneity. Pooled subgroup data by only including studies that used a threshold of SUV_max_ ≥ 5 showed a similar sensitivity of 0.89 (95% CI, 0.83-0.93) but lower specificity of 0.48 (95% CI, 0.23-0.74).

**Figure 5.**
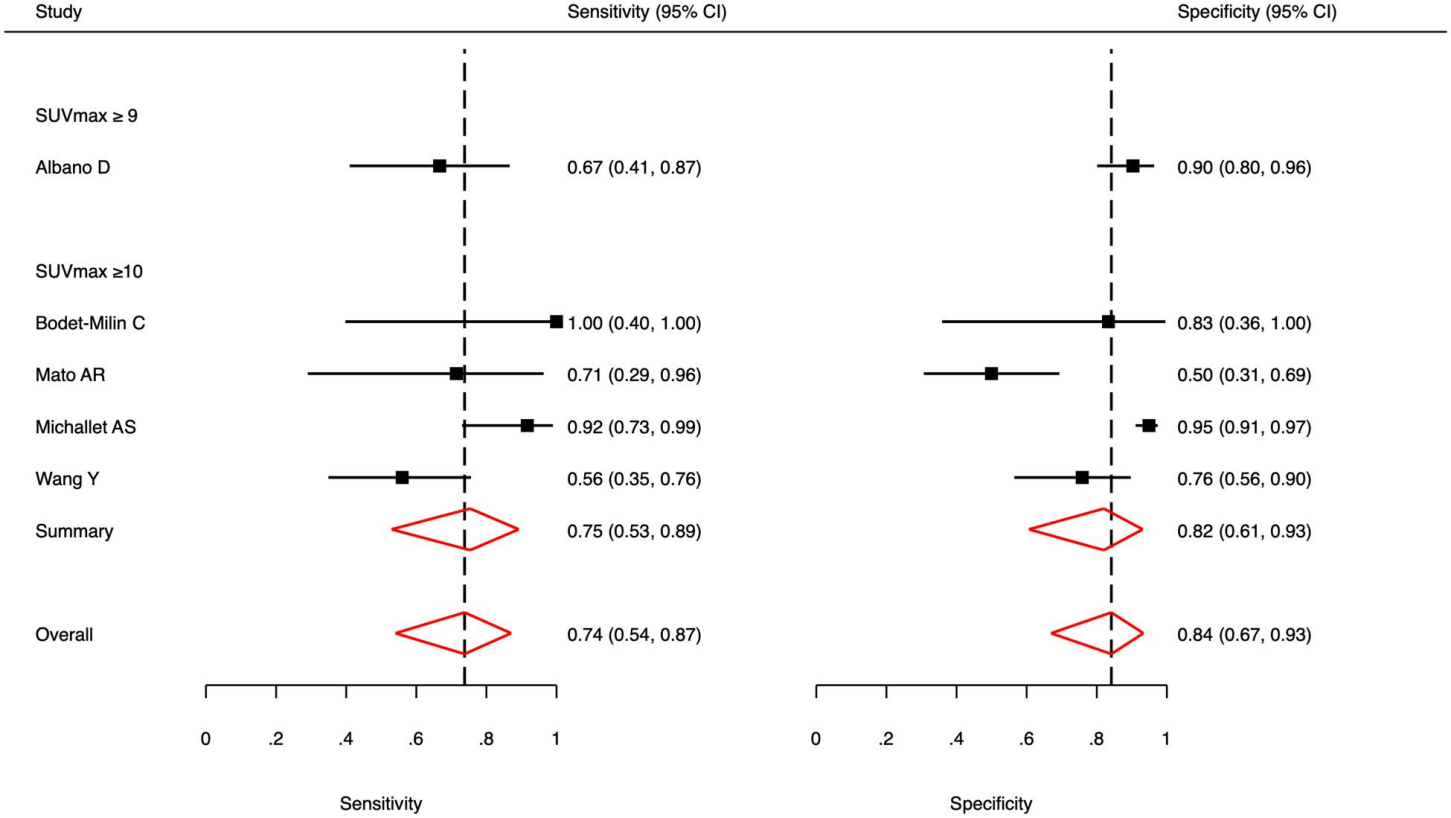
Forest plot of pooled sensitivity and specificity of ^18^F-FDG PET or PET/CT to detect Richter’s transformation using a high SUV_max_ threshold. Heterogeneity (*I^2^*) was 40% for sensitivity and 64% for specificity.

**Figure 6.**
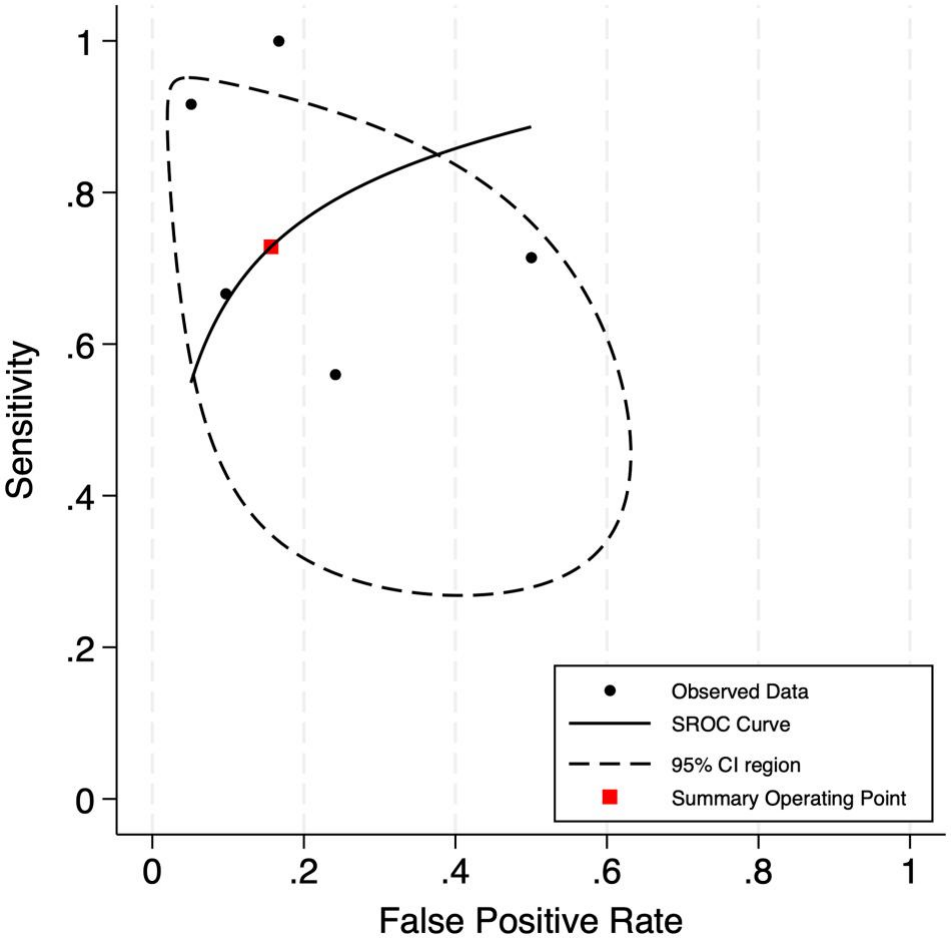
Summary receiver operating characteristic (SROC) curve illustrating the diagnostic accuracy of ^18^F-FDG PET or PET/CT to detect Richter’s transformation using a high SUV_max_ threshold. Area under the curve (AUC) was 0.84.

Forest plots for sensitivity and specificity of the high SUV_max_ group are shown in Figure 5. The pooled sensitivity was 0.74 (95% CI, 0.54-0.87) with heterogeneity (*I^2^*) moderate at 40%. The pooled specificity was 0.84 (95% CI, 0.67-0.93) with heterogeneity (*I^2^*) high at 64%. The SROC curve is illustrated in Figure 6. AUC was 0.84, indicating good diagnostic accuracy but slightly worse than the moderate SUV_max_ group. The correlation coefficient between sensitivity and specificity was 0.352, which suggests high heterogeneity. Pooled subgroup data by only including studies that used a threshold of SUV_max_ ≥ 10 showed a similar sensitivity of 0.75 (95% CI, 0.53-0.89) and specificity of 0.82 (95% CI, 0.61-0.93).

Findings from conference abstracts were suitable only for the moderate SUV_max_ group analysis. The data reported by Zhang et al^22^ and Zheng et al^20^ were not unique, so a separate analysis excluding their findings was not performed. Du^23^ did not report an SUV_max_, and their data were ineligible for meta-analysis.

#### FL and mixed histological populations

High study variability and insufficient evidence prevented a meta-analysis of findings for either the FL or mixed histological population groups. Several SUV_max_ thresholds were reported as most optimal. Data from Bodet-Milin et al^19^ and Wondergem et al^24^ showed that a cut-off of SUV_max_ ≥ 14 and SUV_max_ ≥ 14.5, respectively, were the most effective in detecting HT in FL patients. Rajamäki et al^25^ report the highest cut-off of 26.5; however, they only chose to biopsy FL patients with an SUV_max_ ≥ 10, excluding individuals with SUV_max_ < 10 when calculating diagnostic performance. In mixed populations, Bodet-Milin et al^19^ found an SUV_max_ ≥ 14 to give a sensitivity of 0.94 (95% CI, 0.71-1.00) and specificity of 0.95 (95% CI, 0.76-1.00), while Wai et al^26^ found an SUV_max_ ≥ 15 to give a sensitivity of 0.45 (95% CI, 0.27-0.64) and specificity of 0.74 (95% CI, 0.60-0.85).

## Discussion

To our knowledge, this is the first systematic review and meta-analysis that evaluates the ability of ^18^F-FDG PET or PET/CT to detect HT for different histological subtypes of indolent lymphomas, especially CLL/SLL and FL. Our meta-analysis of 10 studies assessing the performance of ^18^F-FDG PET/CT in detecting Richter’s transformation demonstrates a moderate SUV_max_ threshold (5-6.4) to yield a pooled sensitivity of 0.90, pooled specificity of 0.54, and AUC of 0.89. A high SUV_max_ threshold (9-10) yields a pooled sensitivity of 0.74, pooled specificity of 0.84, and AUC 0.84. Our systematic review of 5 studies assessing the performance of ^18^F-FDG PET/CT for detecting HT in FL or cohorts with mixed histological subtypes found suggested thresholds to range from an SUV_max_ of 10 to 26.5, with varying sensitivities and specificities.

^18^F-FDG PET/CT has several clinical applications in the management of patients with NHLs, including staging, prognostication, treatment monitoring, and detecting recurrence.^5^ If biopsy is feasible, our findings advise that a cut-off of SUV_max_ ≥ 5 would be best for selecting sites for biopsy in patients with CLL/SLL clinically suspicious for Richter’s transformation, since it provides the highest sensitivity and a reasonable FP rate. If biopsy is infeasible, we recommend using a higher cut-off of SUV_max_ ≥ 10, since it provides greater specificity to identify Richter’s transformation and can guide treatment for these patients. Both cut-offs demonstrated high SROC AUC values and offer value depending on clinical context.

A previous systematic review and meta-analysis examined the ability of ^18^F-FDG PET/CT in detecting Richter’s transformation in CLL/SLL patients.^27^ They showed that a threshold of SUV_max_ ≥ 5 results in a pooled sensitivity of 0.87 (95% CI, 0.79-0.93) and a specificity of 0.48 (95% CI, 0.27-0.70) to detect Richter’s transformation. Our findings include more data and support their results, as we demonstrate a pooled sensitivity of 0.89 (95% CI, 0.83-0.93) and specificity of 0.48 (95% CI, 0.23-0.74) when using a threshold of SUV_max_ ≥ 5. When using a threshold of SUVmax ≥ 10, we further demonstrate that a higher specificity is achieved (0.82 [95% CI, 0.61-0.93]), at the cost of reduced sensitivity (0.75 [95% CI, 0.53-0.89]).

The data from studies focusing on FL or populations with mixed histological subtypes were limited and failed to make significant conclusions on PET/CT diagnostic ability. For example, Rajamäki et al^25^ focused on the clinical impact of ^18^F-FDG PET/CT to guide biopsies in patients with FL and found that for those with an SUV_max_ ≥ 10, an SUV_max_ ≥ 26.5 was most optimal in detecting HT. Although they note HT is possible in lower SUV_max_ values, their findings cannot be generalized to determine diagnostic accuracy. Bodet-Milin et al,^19^ Obeid et al,^28^ and Wai et al^26^ identified SUV_max_ cut-offs of 14, 10, and 12, respectively, to be most optimal in detecting HT with good sensitivity for populations with different histological subtypes of indolent lymphomas. Although FL was the most prevalent subtype in these cohorts, combining populations could contribute to significant heterogeneity, as we identify that there were differences in average SUV_max_ among the included studies for CLL/SLL, FL, and mixed histopathologies. Previous studies have also highlighted that MZL and CLL/SLL present with low-to-moderate FDG uptake^11^^,^^19^^,^^29^^,^^30^ while FL can present with FDG uptake higher than the other types of indolent NHLs.^6^^,^^11^^,^^19^

In addition to the detection of HT, ^18^F-FDG PET/CT could have a prognostic role in predicting HT risk in patients with FL. Xie et al^31^ found that a baseline SUV_max_ ≥ 14.3 was highly predictive of a shorter time to HT in a cohort of 219 patients with low-grade or high-grade FL. However, Mir et al^32^ could not demonstrate the same predictive ability of PET/CT in a cohort of patients with FL from the phase 3 GALLIUM study. Their findings show a median baseline SUV_max_ of 12.4 in patients with HT and 11.8 in patients without HT, but a limited population of including only patients with high tumour burden that were eligible for immunotherapy and having only 15 out of 549 patients experience HT may have affected their results. Better elucidation of the predictive ability of pre-transformation PET/CT may present us with a valuable tool to screen for HT in indolent lymphomas, especially since there are no reliable clinical factors identified at initial diagnosis that can predict HT.^33^^,^^34^

Most NHLs are FDG-avid, and SUV remains the most widely used PET parameter to evaluate NHLs because it is directly associated with lymphoma aggressiveness.^35^ Other PET parameters such as metabolic tumour volume (MTV) and total lesion glycolysis (TLG) have been examined for predictive and diagnostic ability. Previous studies showed their prognostic value in FL and CLL.^36^^,^^37^ For detection, Albano et al^21^ could not demonstrate a role of either MTV or TLG, but Obeid et al^28^ found high TLG to detect HT in a mixed histological subtype population. Further exploration of the role of other metabolic parameters in conjunction with SUV may introduce methods by which HT of NHLs can be better characterized.

A few limitations affect our systematic review and meta-analysis. We include a small sample size, and most eligible studies were conducted retrospectively. Not all studies incorporated a cohort design, which is preferred for diagnostic accuracy analyses.^38^ Discrepancies in methodology and clinical characteristics of patients, notably histological subtype, lymphoma grade, and prior treatment, contribute to the high heterogeneity between individual studies. Since fewer than 10 studies were included in the meta-analysis, meta-regression for subgroup analysis to identify potential causes of heterogeneity was not advised^39^ but could have provided valuable information if more evidence was available.

In summary, ^18^F-FDG PET/CT demonstrates good diagnostic accuracy to detect Richter’s transformation when using a moderate threshold of SUV_max_ ≥ 5. A high threshold of SUV_max_ ≥ 10 yields slightly lower diagnostic accuracy with greater specificity at the cost of slightly lower sensitivity. SUV_max_ thresholds may be limited in discriminating FL from HT, and alternatives should be sought. Limited evidence, especially surrounding FL, suggests the need for larger, more robust studies to better elucidate the diagnostic accuracy of ^18^F-FDG PET/CT or explore other methods to detect HT in indolent lymphomas.

## Supplementary Material

tqaf028_Supplementary_Data
